# BTG1 potentiates apoptosis and suppresses proliferation in renal cell carcinoma by interacting with PRMT1

**DOI:** 10.3892/ol.2015.3293

**Published:** 2015-05-29

**Authors:** CHUNHUI LIU, TAO TAO, BIN XU, KAI LU, LEI ZHANG, LIANG JIANG, SHUQIU CHEN, DACHUANG LIU, XIAOWEN ZHANG, NIHAO CAO, MING CHEN

**Affiliations:** 1 Department of Urology, Affiliated Zhongda Hospital of Southeast University, Nanjing, Jiangsu 210009, P.R. China; 2 Department of Urology, Haimen City People's Hospital, Haimen, Jiangsu 226100, P.R. China

**Keywords:** renal cell carcinoma, B-cell translocation gene 1, protein arginine N-methyltransferase 1, cell cycle, apoptosis, proliferation

## Abstract

B-cell translocation gene 1 (BTG1) is a member of the BTG/transducer of Erb family. BTG1 regulates cell cycle progression, inhibits proliferation, promotes apoptosis and stimulates cellular differentiation in multiple cell types. However, the functions of BTG1 in renal cell carcinoma (RCC) remain unclear. Therefore, the present study investigated the role of BTG1 in RCC tissue samples and 786-O RCC cells. RCC tissues and cells exhibited significantly weaker BTG1 protein and mRNA expression compared with para-carcinoma control tissues (P<0.05). Upregulated BTG1 expression induced significant G0/G1 cell cycle arrest, apoptosis and inhibition of cell proliferation in 786-O cells (P<0.05). Furthermore, BTG1 interacted with protein arginine N-methyltransferase 1 (PRMT1), and blocking the action of PRMT1 in 786-O cells resulted in inhibition of BTG1 function. These findings indicate that BTG1 may inhibit cell growth and promote apoptosis by interacting with PRMT1 in RCC; the identification of this mechanism may aid in the production of novel therapies for RCC.

## Introduction

Renal cell carcinoma (RCC) is among the most common types of cancer and accounts for ~2–3% of all malignancies (1). The incidence of RCC continuously increases each year, with an estimated 61,560 new cases of RCC and 14,080 RCC-associated mortalities predicted to occur in the United States in 2015 (2). Despite advances in diagnostic techniques, 25–30% of patients present with metastatic disease (3). The prognosis is poor for such patients, with subsequent chemotherapy and radiotherapy treatment regimes yielding ineffective results (4). Tumourigenesis and progression are multistep processes that are affected by changes in gene expression. Therefore, understanding the gene expression changes that occur in RCC may improve the diagnosis, treatment and prevention of RCC.

B-cell translocation gene 1 (BTG1) is a member of the BTG/transducer of Erb (TOB) family. This family comprises six members; BTG1, BTG2/TIS21/PC3, BTG3, BTG4/PC3B, TOB1 and TOB2. The BTG/TOB family proteins are composed of two highly conserved and characteristic domains, Box A and Box B, in the N-terminal region. In addition, these proteins are involved in regulating cell cycle progression, inhibiting proliferation, promoting apoptosis and stimulating cellular differentiation in multiple cell types (5). As BTG1 exhibits these characteristics, it is considered to be a tumour suppressor gene (6). Previous studies have identified that BTG1 enhances the antiproliferative function of homeobox B9-mediated transcription (7), while overexpression of BTG1 induces increased apoptosis in NIH 3T3 cells (8). However, the functions of BTG1 and its precise molecular mechanisms in RCC remain unclear. It has been shown that BTG1 interacts with protein arginine N-methyltransferase 1 (PRMT1) *in vitro* (9). PRMT1 then catalyses the formation of ω-monomethylarginine and asymmetric dimethylarginine. This arginine methylation regulates transcription or affects cytokine signalling pathways (10). However, whether BTG1 functions in RCC via its effect on PRMT1 remains unknown.

Therefore, the present study examined BTG1 expression in RCC tissues and cells, and investigated the function of BTG1 in cell proliferation, cell cycle distribution and apoptosis *in vitro*. In addition, it was investigated whether the functions of BTG1 are attributable to interactions with PRMT1.

## Materials and methods

### 

#### Tissues

RCC and corresponding para-carcinoma tissue samples were obtained from 20 patients, who underwent nephrectomy at the Affiliated Zhongda Hospital of Southeast University (Nanjing, China) between June 2007 and June 2010. Histological diagnoses were established following analysis of standard hematoxylin and eosin-stained sections by two senior pathologists experienced in RCC diagnosis. All patients were diagnosed with RCC. Approval was obtained from the ethics committee of the Affiliated Zhongda Hospital of Southeast University and samples were collected following receipt of written informed consent from all patients.

#### Reverse transcription-quantitative polymerase chain reaction (RT-qPCR)

Total RNA was extracted from the specimens using TRIzol® reagent (Invitrogen Life Technologies, Carlsbad, CA, USA). Complementary (c)DNA synthesis was performed using Avian Myeloblastosis Virus Reverse Transcriptase and random primers (Takara Biotechnology Co., Ltd., Dalian, China), and the RT-qPCR reactions were performed using a 7300 Real-Time RT-PCR system (Applied Biosystems Life Technologies, Foster City, CA, USA). The amplification steps consisted of 95°C for 1 min, 40 cycles at 95°C for 15 s and 60°C for 30 s, followed by 72°C for 10 min. The primer sequences for BTG1 were as follows: F 5′-ATCTCCAAGTTTCTCCGCACC-3′ and R 5′-CAACGGTAACCCGATCCCTT-3′. Subsequently, the expression BTG1 mRNA was calculated relative to GAPDH mRNA expression levels using the 2^−ΔΔCt^ method.

#### Immunohistochemistry

All surgical samples were fixed in 10% buffered formaldehyde solution and embedded in paraffin. Paraffin sections (4-µm thick) were then reacted with monoclonal antibodies against BTG1 (mouse anti-human; 1:75 dilution; cat. no. ab50991; Abcam, Cambridge, UK). The antibody was replaced by phosphate-buffered saline (PBS) as a negative control.

#### Immunohistochemical evaluation

The BTG1 immunohistochemistry results were scored using semi-quantitative immunoreactivity scores (IRSs) for all sections. The intensity of staining and percentage of positively-stained cells were noted. The intensity of staining was scored as follows: No staining, 0; mild staining, 1; moderate staining, 2; and strong staining, 3. The percentage of positive cells was scored as follows: No positive cells, 0; <5% positive cells 1; 5–25% positive cells, 2; 26–50% positive cells, 3; and >50% positive cells, 4. The overall IRSs were calculated as follows: IRS = percentage of positive cells × intensity of staining. Expression was then classified as negative (–; IRS, 0), weak (positive, +; IRS, 1–2), moderate (double positive, ++; IRS, 3–4) or strong (triple positive, +++; IRS, 4–12).

#### Plasmid construction

cDNA encoding human BTG1 was generated by RT-PCR and subcloned into the EcoRI and MluI restriction sites of the pCI-neo expression vector (Promega Corporation, Madison, Wisconsin, USA). The plasmid sequence was confirmed using sequencing.

#### Cell culture and transfection

The 786-O and HK-2 cells were obtained from the Cell Resource Centre, Institute of Basic Medical Sciences, Chinese Academy of Medical Sciences (Shanghai, China). The cells were cultured in RPMI 1640 (GE Healthcare Life Sciences, Beijing, China) supplemented with 50 U/ml penicillin, 50 mg/ml streptomycin (Generay Biotech Ltd., Shanghai, China) and 10% fetal bovine serum (GE Healthcare Life Sciences, Carlsbad, USA) in an atmosphere of 5% CO_2_ at 37°C. Cells were transfected with the pCI-neo/BTG1 vector using Lipofectamine 2000 (Invitrogen Life Technologies), according to the manufacturer's instructions, and eosin Y disodium trihydrate (Santa Cruz Biotechnology, Inc., Dallas, TX, USA) was added to inhibit PRMT1 in a proportion of the BTG1-overexpressed 786-O cells. The 786-O cells were transfected with blank pCI-neo vector as the control.

#### Western blot

Whole cell extracts (786-O RCC and HK-2 control cells) were separated by 10% SDS-PAGE and transferred onto polyvinylidene fluoride membranes. Antigen retrieval was performed in heated citrate buffer (PH 6.0). Blots were blocked with 5% non-fat milk at room temperature for 1 h and incubated with the monoclonal mouse anti-human BTG1 (1:500 dilution; cat. no. ab50991; Abcam, Cambridge, UK) antibody in blocking buffer overnight at a temperature of 4°C. The blots were then washed and incubated with horseradish peroxidase-labelled goat anti-mouse IgG secondary antibody (1:3,000 dilution; cat. no. ZB-2301; Zhongshan Goldenbridge Biotechnology Co., Ltd., Beijing, China) and visualised using enhanced chemiluminescence (Beyotime Institute of Biotechnology, Haimen, China).

#### Cell viability and clonability assays

Cell viability was determined at 24, 48 and 72 h by performing an MTT assay. In brief, 786-O cells transfected for >48 h were seeded into 96-well plates (3,000 cells/plate), and cultured for 24, 48 and 72 h. Each well was supplemented with 20 µl MTT (5 mg/ml) and incubated at 37°C for 4 h. The supernatant was removed and the purple precipitates of formazan were dissolved in 200 µl DMSO (Sigma-Aldrich, St. Louis, MO, USA). Absorbance was then read at a wavelength of 570 nm using an automatic multi-well MK3 spectrophotometer (Thermo Fisher Scientific, Inc., Waltham, MA, USA). For the clonability assay, cells were counted >48 h after transfection, seeded into 6-well plates at a low density (3,000 cells/plate) and cultured for 9–14 days until visible colonies appeared. Cells were subsequently stained with methyl violet and the number of colonies was counted under observation of a Olympus CKX41 light microscope (Olympus Corporation, Tokyo, Japan).

#### Flow cytometry

Fluorescence-activated cell-sorting analysis was performed 72 h after transfection. The cells were harvested, washed with cold PBS and resuspended. Cells were then stained with propidium iodide (PI) for cell cycle analysis, and PI plus Annexin-V-fluorescein isothiocyanate (FITC) for apoptosis analysis using an Annexin V-FITC/PI Apoptosis kit (UBio Biological Technology Co., Ltd, Ji'nan, China), according to the manufacturer's instructions.

#### Co-immunoprecipitation

Total proteins were extracted from 786-O cells using cell lysis buffer for western blotting and immunoprecipitation (Beyotime Institute of Biotechnology) was performed using protease inhibitors (Amresco LLC, Solon, OH, USA). The primary antibody used was rabbit anti-human PRMT1 polyclonal antibody (1:100 dilution, cat. no. sc-130851; Santa Cruz Biotechnology, Inc.) and the secondary antibody used was mouse anti-BTG1 monoclonal antibody (Abcam). For co-immunoprecipitation, total cellular protein was incubated with 10 µl anti-PRMT1 antibody for 1 h at 4°C. Subsequently, 20 µl Protein A/G PLUS-Agarose was added and the capped tubes were rocked overnight at 4°C. Beads were then pelleted by centrifugation at ~600 × g. The supernatant was removed, and the beads were washed, boiled in 1X SDS sample buffer and subjected to SDS-PAGE gel electrophoresis and western blotting with the anti-BTG1 antibody.

#### Immunostaining and confocal laser microscopic analysis

Cells were grown in culture dishes with a bottom well. Monolayers were washed with PBS, fixed with immunostaining fix solution (Beyotime Institute of Biotechnology) for 1 h at routine temperature, washed with Tris-buffered saline and Triton X-100 (2.42 g Tris, 8 g NaCl, 0.1% Triton X-100) three times, and blocked with immunostaining blocking buffer (Beyotime Institute of Biotechnology) for 1 h. The monolayers were incubated overnight with the BTG1 and PRMT1 antibodies at a temperature of 4°C. After washing, cells were incubated with cyanine 3-conjugated anti-rabbit antibody and FITC-conjugated anti-mouse antibody (Beyotime Institute of Biotechnology) for 1 h at a routine temperature. Finally, specimens were examined under a confocal laser scanning microscope (LSM 710; Zeiss, Oberkochen, Germany).

#### Statistical analysis

All quantitative data represent a mean value of at least triplicate samples and all statistical analyses were performed using Statistical Package of the Social Sciences software (version 16.0; SPSS, Inc., Chicago, IL, USA). Data are presented as the mean ± standard error of the mean. All calculations were performed using GraphPad Prism 5 software (GraphPad Software, Inc., La Jolla, CA, USA) and scanned images of western blots were quantified using ImageJ 2X software (National Institutes of Health, Bethesda, MD, USA). Group means were compared by performing Student's t-test. P<0.05 was considered to indicate a statistically significant difference.

## Results

### 

#### Low BTG1 expression in RCC tissues and cells

**Figure 1.**
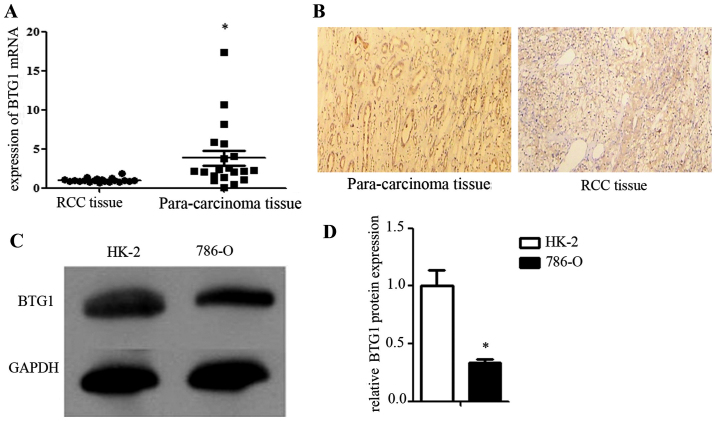
BTG1 expression in RCC. (A) Reverse transcription-quantitative polymerase chain reaction and (B) and immunohistochemistry demonstrating low BTG1 expression levels in RCC. *P<0.05, vs. para-carcinoma tissue. (C) Representative western blot and (D) quantified western blotting data, demonstrating low BTG1 expression in RCC cells. *P<0.05, vs. HK-2 control cells. BTG1, B-cell translocation gene 1; RCC, renal cell carcinoma.

In order to investigate BTG1 expression in RCC, BTG1 mRNA expression was measured using RT-qPCR, and BTG1 protein expression was determined using immunohistochemistry, in 20 RCC and 20 corresponding para-carcinoma tissue samples. BTG1 expression was significantly lower in the RCC tissues compared with that in the corresponding para-carcinoma tissues (P<0.05; Fig. 1A and 1B). In order to construct a reliable *in vitro* model for investigating the mechanism of action of BTG1 in RCC, BTG1 expression was examined by western blot analysis in HK-2 (control) and 786-O (RCC) cells. BTG1 expression was significantly lower in the 786-O cells compared with that in HK-2 cells (P<0.05; Fig. 1C and 1D).

#### In vitro effects of BTG1 in RCC

**Figure 2.**
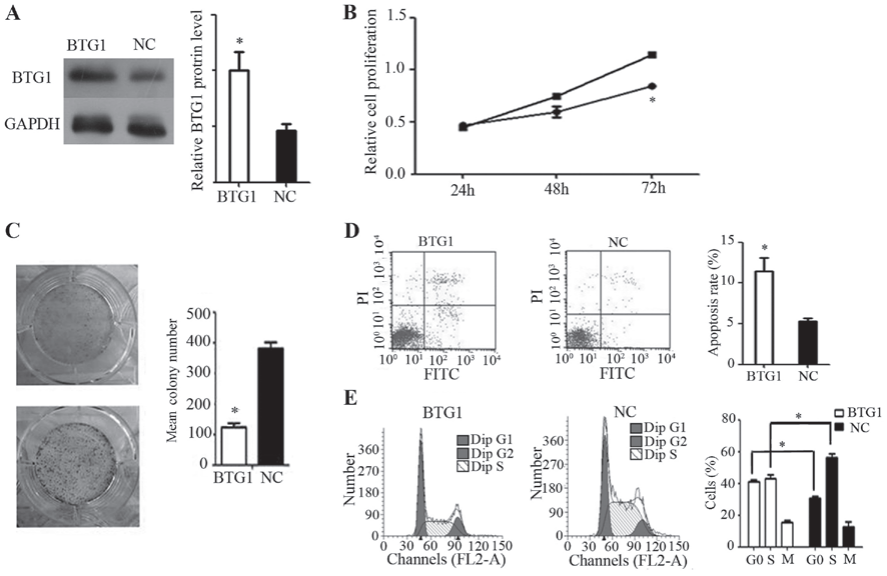
Effect of BTG1 on 786-O cells *in vitro*. (A) Western blotting demonstrated increased BTG1 expression following transfection. *P<0.05, vs. NC. (B) Cell proliferation rate, showing that BTG1 overexpression significantly inhibited 786-O proliferation, as determined by an MTT assay. *P<0.05, vs. cells not overexpressing BTG1. (C) Colony forming assay, demonstrating reduced colony-forming efficiency in 786-O cells with forced BTG1 expression. *P<0.05, vs. NC. (D) Flow cytometry, demonstrating a higher rate of apoptosis in 786-O cells with forced BTG1 expression, as evaluated by flow cytometry. *P<0.05, vs. NC. (E) Cell cycle analysis of BTG1-overexpressing 786-O cells, showing an increase in the proportion of cells in the G0/G1 phase. *P<0.05. BTG1, B-cell translocation gene 1; NC, negative control; PI, propidium iodide.

In order to investigate the function of BTG1 in RCC, a BTG1 overexpression plasmid was transfected into 786-O cells. Western blot analysis identified a significant increase in BTG1 protein expression in the 786-O cells following transfection (P<0.05; Fig. 2A). Additionally, BTG1 overexpression significantly inhibited 786-O cell proliferation, as shown by MTT and colony formation assays (P<0.05; Fig. 2B and 2C). Furthermore, flow cytometry assays revealed a significant increase in cell apoptosis and G0/G1 arrest in 786-O cells, following transfection (P<0.05; Fig. 2D and 2E).

#### BTG1 protein functions via PRMT1

**Figure 3.**
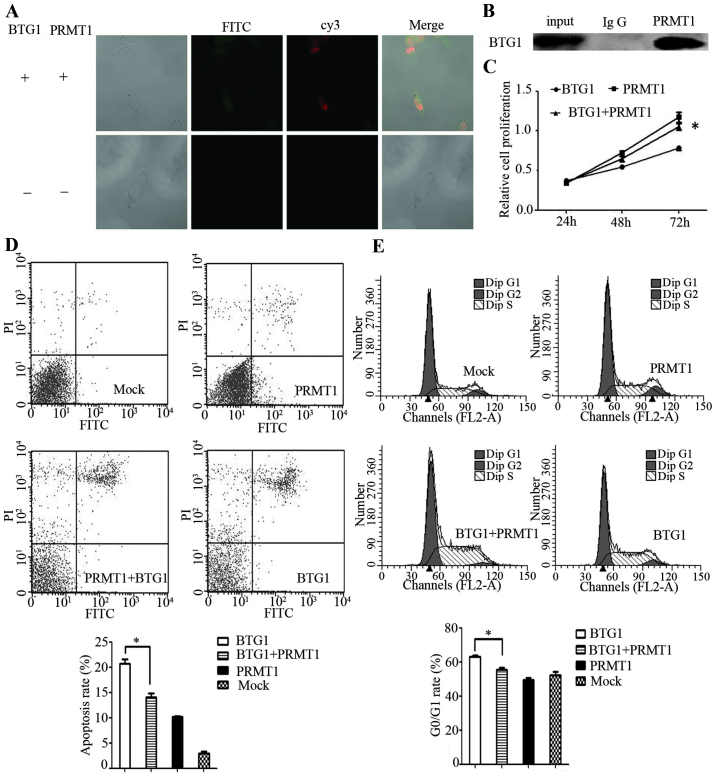
BTG1 protein functions via PRMT1. (A) and (B) BTG1 interacts with PRMT1 in 786-O cells. (C) MTT assay, demonstrating that the effect of BTG1 in inhibiting proliferation was induced by PRMT1 inhibition. (D) The effect of BTG1 on apoptosis was suppressed by PRMT1 inhibition. (E) Blockage of the cell cycle induced by BTG1 was suppressed by PRMT1 inhibition. *P<0.05. BTG1, B-cell translocation gene 1; PRMT1, protein arginine N-methyltransferase 1; FITC, fluorescein isothiocyanate; PI, propidium iodide.

In order to clarify whether BTG1 functions via PRMT1, the interactions between BTG1 and PRMT1 were demonstrated by immunofluorescence and co-immunoprecipitation experiments in 786-O cells. BTG1 interacted with PRMT1 in 786-O cells (Fig. 3A and 3B). Eosin Y disodium trihydrate was added to inhibit PRMT1 in BTG1-overexpressing 786-O cells. This inhibition resulted in significantly reduced proliferation, G0/G1 phase arrest and apoptosis in the BTG1-overexpressing 786-O cells (P<0.05; Fig. 3C–E), indicating that BTG1 may function by interacting with PRMT1.

## Discussion

BTG1 was initially identified in B lymphoblastic leukaemia and its expression appears to be highest in the G0/G1 phases of the cell cycle (11). Weak BTG1 expression has recently been reported in various other types of cancer, including thyroid, lung and breast (12–15). In the present study, weak BTG1 expression was also demonstrated in RCC tissue samples by performing qRT-PCR, western blotting and immunohistochemistry.

Tumour development and progression are associated with uncontrolled proliferation and apoptosis. Zhu *et al* (16) and Sun *et al* (17) identified that BTG1 induces G0/G1 cell cycle arrest, promotes apoptosis and inhibits cell proliferation in breast and non-small cell lung cancer (NSCLC), respectively. Furthermore, Sun *et al* (18) determined that BTG1 expression was significantly correlated with lymph node metastasis, clinical stage, histological grade and survival in NSCLC cells. The present study explored the functions of BTG1 in RCC, and obtained similar results, with respect to the effect of BTG1 expression on cell cycle arrest, apoptosis promotion and growth inhibition. These data indicate that BTG1 may have a common antitumour function in various types of cancer.

The antiproliferative activity of BTG1 is controlled by the conserved Box A domain (11). Doidge *et al* (18) demonstrated that the antiproliferative activity of BTG1 is mediated through its interactions with the Caf1a and Caf1b deadenylase enzymes, and the role of BTG1 in the regulation of mRNA abundance and translation is dependent on Caf1a/Caf1b. However, in the present study, BTG1 appeared to function by interacting with PRMT1. In mammals, PRMT1 is the primary arginine asymmetric dimethylation enzyme, accounting for >90% of asymmetric dimethylation enzymes (19). Arginine methylation is a common post-translational modification that alters the stability of chromatin and affects the binding of transcriptional factors, thereby regulating gene expression without changing the original nucleotide sequence. Arginine methylation has critical functions in gene transcription, mRNA splicing, DNA repair, protein cellular localisation and signalling (20), and PRMT1 specifically exhibits key functions in breast cancer cell apoptosis and osteosarcoma cell proliferation (21–23). The present study investigated the interaction between BTG1 and PRMT1 in RCC, and it was shown that certain functions of BTG1 are suppressed by the inhibition of PRMT1. Thus, it is hypothesized that BTG1 functions by interacting with PRMT1 in RCC, as well as through other signalling pathways.

In conclusion, the present study demonstrated weak expression of BTG1 in RCC, and showed that BTG1 may inhibit cell growth and promote cell apoptosis by interacting with PRMT1. However, the underlying mechanism of action of PRMT1 in RCC requires further investigation.
